# Fractures in women treated with raloxifene or alendronate: a retrospective database analysis

**DOI:** 10.1186/1472-6874-13-15

**Published:** 2013-03-23

**Authors:** Shonda A Foster, Nianwen Shi, Suellen Curkendall, John Stock, Bong-Chul Chu, Russel Burge, David R Diakun, John H Krege

**Affiliations:** 1 Eli Lilly and Company, Indianapolis, IN, USA; 2 Truven Health Analytics, Bethesda, MD, USA; 3 Lilly USA, LLC, Indianapolis, IN, USA

**Keywords:** Vertebral fracture, Nonvertebral fracture, Breast cancer, Alendronate, Raloxifene

## Abstract

**Background:**

Raloxifene and alendronate are anti-resorptive therapies approved for the prevention and treatment of postmenopausal osteoporosis. Raloxifene is also indicated to reduce the risk of invasive breast cancer in postmenopausal women with osteoporosis and in postmenopausal women at high risk of invasive breast cancer. A definitive study comparing the fracture effectiveness and rate of breast cancer for raloxifene and alendronate has not been published. The purpose of this retrospective cohort study was to evaluate fracture and breast cancer rates among patients treated with raloxifene or alendronate.

**Methods:**

Females ≥45 years who initiated raloxifene or alendronate in 1998–2006 Truven Health Analytics *MarketScan® Databases*, had continuous enrollment 12 months prior to and at least 12 months after the index date, and had a treatment medication possession ratio ≥80% were included in this study. Rates of vertebral and nonvertebral fractures and breast cancer during 1, 3, 5, 6, 7, and 8 years of treatment with raloxifene or alendronate were evaluated. Fracture rates were adjusted for potential treatment bias using inverse probability of treatment weights. Multivariate hazard ratios were estimated for vertebral and nonvertebral fractures.

**Results:**

Raloxifene patients had statistically significantly lower rates of vertebral fractures in 1, 3, 5, and 7 years and for nonvertebral fractures in 1 and 5 years. There were no statistically significant differences in the adjusted fracture rates between raloxifene and alendronate cohorts, except in the 3-year nonvertebral fracture rates where raloxifene was higher. Multivariate hazard ratios of raloxifene versus alendronate cohorts were not significantly different for vertebral and nonvertebral fracture in 1, 3, 5, 6, 7, and 8 years. Unweighted and weighted breast cancer rates were lower among raloxifene recipients.

**Conclusions:**

Patients treated with alendronate and raloxifene had similar adjusted fracture rates in up to 8 years of adherent treatment, and raloxifene patients had lower breast cancer rates.

## Background

Osteoporosis is the most common bone disease in the United States, affecting approximately 10 million Americans with an additional 34 million people at risk [1]. Osteoporosis contributes to more than 1.5 million fractures each year and is the primary underlying cause of fractures in the elderly [2]. Millions of individuals are at risk for fracture due to risk factors such as low bone mass and advanced age [1,3]. It is expected that in 2025 there will be as many as 3 million osteoporotic-related fractures in the United States [4].

Alendronate and raloxifene are anti-resorptive therapies approved for the prevention and treatment of postmenopausal osteoporosis. Alendronate is incorporated into the bone matrix and acts to inhibit osteoclasts [5,6]. Raloxifene binds to estrogen receptors and appears to act as an estrogen agonist in bone [7]. Both drugs reduce bone turnover and increase bone mineral density, though alendronate has a stronger effect on these domains than raloxifene [8]. Raloxifene is also indicated to reduce the risk of invasive breast cancer in postmenopausal women with osteoporosis and in postmenopausal women at high risk of invasive breast cancer [9,10]. Raloxifene is not indicated for the treatment of invasive breast cancer, reduction of the risk of recurrence of breast cancer, or reduction of the risk of noninvasive breast cancer. Although both therapies have established efficacy from randomized clinical trials, comparative, real-world evidence on these therapies for post-menopausal women may provide important information for health care providers to supplement the clinical trial evidence.

A definitive study comparing the fracture effectiveness of raloxifene and alendronate has not been published [11]. The EVA trial (Evista Alendronate Comparison trial) was designed to be the first double-blind, randomized comparison trial to compare osteoporosis therapies head-to-head for fracture risk reduction among 3,000 postmenopausal women [11]. However, the study enrollment was stopped early due to difficulties with timely recruitment of treatment naïve women. Only 122 patients reached the 2-year endpoint, and the statistical power became too low for establishing non-inferiority between drugs for fracture risk reduction.

We conducted a retrospective database study examining patients who initiated raloxifene or alendronate and were adherent to treatment during 6 time periods. Vertebral and nonvertebral fracture rates were compared during each respective treatment period. Due to the clinical importance of breast cancer risk reduction in some postmenopausal women with osteoporosis, we also compared breast cancer rates in the raloxifene and alendronate cohorts. However, we did not specifically examine the rates of invasive breast cancer versus the rates of recurrent breast cancer or noninvasive breast cancer.

## Methods

### Data source

Study samples were drawn from 1998 to 2008 Truven Health Analytics MarketScan® Commercial Claims and Encounters (CCAE) and Medicare Supplemental and Coordination of Benefits (COB) Databases (MDCR). Both databases contain de-identified health insurance enrollment information and claims data for inpatient and outpatient medical services as well as outpatient prescriptions. Enrollee demographic information is available and medical claims include key data elements such as service date, treatment setting, diagnosis, procedure, and provider type. The CCAE database includes employees, spouses, and dependents covered by employer-sponsored private health insurance. The MDCR database profiles the healthcare experience of retirees with Medicare supplemental insurance paid by employers. Together, both databases consist of an average of 19 million lives annually during the study period of 1998 to 2008. The patient data used in this analysis were de-identified in compliance with the Health Insurance Portability and Accountability Act (HIPAA) regulations; thus, the study did not require Institutional Review Board approval.

### Patient selection

**Figure 1:**
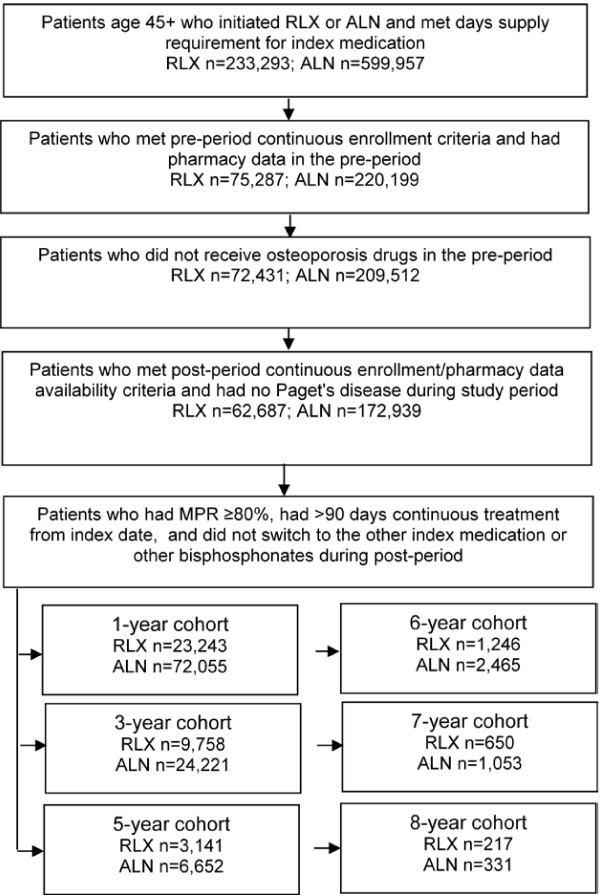
**Patient sample selection.** ALN=alendronate; MPR=medication possession ratio; RLX=raloxifene.

Female patients who initiated raloxifene or alendronate between 1/1/1998 and 12/31/2006 were identified. The index date was the first prescription date of raloxifene or alendronate and patients were assigned to a treatment cohort based on their initial prescription. Patients were excluded if they were under age 45 at index or did not have 12 months of continuous enrollment prior to and minimum of 12 months of continuous enrollment following the index date. Patients were required to have days supply of 1–180 on their initial prescription, have no osteoporosis medications in the pre-period, and no osteoporosis medications other than the index medication during the post-period. Patients with Paget’s disease during the pre- or post-periods were excluded. Patients were followed from 12 months prior to treatment initiation until the end of insurance eligibility or study end (June 30, 2008), whichever came first. Medication possession ratio (MPR) was evaluated for the follow-up periods starting from treatment initiation. Since we were interested in both early and later changes during treatment with raloxifene and alendronate, we measured MPR in 1-, 3-, 5-, 6-, 7-, and 8-year cohorts. Later changes were measured more frequently due to the anticipated smaller sample sizes as the follow-up period lengthened. Patients who had MPR of 80% or higher on their index medication during the follow-up period and at least 90 days of continuous treatment from the index date were considered adherent and included in the final study samples (Figure 1).

### Outcomes

The study’s primary endpoints were the comparative vertebral and nonvertebral fracture rates (i.e. the percent of patients having a fracture) among patients with 1, 3, 5, 6, 7, and 8 years of adherent treatment with raloxifene versus alendronate. Fracture rates were compared between treatment groups during 6 separate observation periods. Multivariate models were used to estimate the hazard ratios of fractures in the 6 cohorts. Additionally, a sensitivity analysis excluding patients with any pre-index fracture diagnosis was performed in the 3-year cohort.

The rate of breast cancer (i.e. the percent of patients having breast cancer) during 1, 3, 5, 6, 7, and 8 years of treatment with raloxifene versus alendronate was also evaluated. A sensitivity analysis was conducted excluding pre-index breast cancer.

Fracture was identified by primary or secondary diagnosis (International Classification of Disease, 9th Edition, Clinical Modification [ICD-9-CM]) indicating a fracture on a non-diagnostic claim of inpatient, emergency room, or outpatient service. Diagnostic claims, such as lab or radiology claims, were excluded since they do not conclusively confirm the presence of a fracture. Fractures due to trauma, identified either by an E code for motor vehicle accident on the same claim, or by 3 or more different fractures within 7 days before or after the fracture of interest, were excluded. Additionally, open fractures were excluded because they are more likely to be related to trauma rather than osteoporosis [12]. Pathological fractures were included if patients were verified as not having cancer prior to and within 6 months after the pathological fracture diagnosis was observed. This is consistent with the recommendation by Curtis and colleagues to include pathological fractures in epidemiologic studies of osteoporotic fractures [13]. In addition, stress fractures were included. In order to appropriately identify treatment-emergent fractures, fractures occurring during the first 90 days of follow-up were excluded. This was similar to the method Meijer and colleagues used in assessing the impact of compliant osteoporosis treatment on fracture risk [14].

The occurrence of fracture during the post-period was identified and fracture rates were calculated for clinical vertebral fracture and nonvertebral fracture (hip, upper leg, lower leg, pelvis, humerus, wrist/forearm, clavicle/rib, and other). Fracture sites were determined by 3-digit ICD-9-CM diagnosis codes. Due to the difficulty of using medical claims to positively identify new fractures at the same site or to distinguish fractures of the right arm/leg from the left, only the first fracture at each of the fracture sites was counted.

Breast cancer was identified by either ICD-9-CM diagnosis codes or ICD-9-CM and CPT procedure codes indicating surgical procedure to treat breast cancer. Binary variables were created to flag the presence of breast cancer in the pre-period and in the 6 follow-up periods. A sensitivity analysis was performed by evaluating breast cancer rates in the post-period in patients without a code indicative of breast cancer in the pre-period.

### Statistical analysis

The results were adjusted for potential confounding using inverse probability of treatment weights (IPTWs) [15-17]. First, a binary logistic regression model was fitted to calculate the propensity score, the probability of a subject initiating raloxifene versus alendronate, conditional on the observed baseline covariates. IPTWs were created using the propensity score: for the raloxifene cohort, the IPTW was [1/propensity score]; for the alendronate cohort, the IPTW was [1/(1 – propensity score)]. The outcomes were weighted by applying the IPTWs to each observation. Covariates in the logistic regression model included demographic characteristics (age, Medicare eligibility, region, urban residence, insurance capitation status, and provider type that was closest to the fill date of index prescription), baseline Deyo Charlson Comorbidity Index, pre-period bone mineral density (BMD) screening or mammography, presence of pre-index fracture, presence of baseline comorbid conditions (HIV/AIDs, liver disease, cancer, osteoporosis, renal disease, rheumatoid arthritis, heart disease, hypertension, gastric disease, vasomotor symptoms, osteonecrosis of the jaw, diabetes, alcoholism, deep venous thrombosis/pulmonary embolism, osteodystrophy, thyroid disease, metabolic disorders, dysphagia, and dyslipidemia), and pre-period use of other medications (glucocorticoids, immunosuppressants, anticonvulsants, estrogen/hormone therapy, hormone deprivation therapy, prescription aspirin/acetaminophen, NSAIDS, opioids, benzodiazepines, and skeletal muscle relaxants).

Statistical tests of significance for differences between raloxifene and alendronate were conducted. Chi-square tests were used to evaluate the statistical significance of differences for categorical variables; independent *t* tests were used for evaluating significance of continuous variables. To assess the validity of propensity score weighting, standardized differences were calculated to assess group balances before and after the weights were applied to the variables entered as covariates in the propensity model [18].

To further adjust for the remaining imbalances between the raloxifene and alendronate cohort, we obtained the hazard ratios for vertebral and nonvertebral fractures in 6 separate observation periods. In the nonvertebral fracture model, patients could potentially have multiple fractures of different sites during the observation period. For example, a patient could have a fracture of the lower leg and then later a hip fracture. Under this circumstance, the marginal proportional hazards model is considered as an appropriate specification [19] and was used to obtain an overall hazard ratio for the hazard of multiple fractures. For the vertebral fracture model, the hazard ratio was obtained using the standard Cox model since we only captured a patient’s first vertebral fracture event due to the challenge of identifying subsequent fracture at the same fracture site using administrative claims. Alendronate served as the reference group in all models. The models were weighted using the IPTWs and the same list of covariates was included in all models: age, pre-index BMD screening or mammography, pre-index use of glucocorticoids, immunosuppressants, anticonvulsants, aspirin/acetaminophen, NSAIDS, opioids, benzodiazepines, skeletal muscle relaxants, pre-index dyslipidemia, rheumatoid arthritis, heart disease, vasomotor symptoms, gastric disease, osteoporosis, and fracture. A sensitivity analysis was conducted which excluded patients with a pre-index fracture diagnosis for the 3-year cohort.

## Results

### Patient characteristics

Six cohorts were identified based on the length of adherent therapy: 1 year (raloxifene [n = 23,243], alendronate [n = 72,055]); 3 years (raloxifene [n = 9,758], alendronate [n = 24,221]); 5 years (raloxifene [n = 3,141], alendronate [n = 6,652]); 6 years (raloxifene [n = 1,246], alendronate [n = 2,465]); 7 years (raloxifene [n = 650], alendronate [n = 1,053]); and 8 years (raloxifene [n = 217], alendronate [n = 331]). Pre-period demographic and clinical characteristics are presented in Table 1. The mean age ranged between 60.7 and 65.3 years. Patients in the raloxifene cohorts were approximately 2 years younger than those in the alendronate cohorts (p < 0.001). The most common pre-period conditions were hypertension, “other cardiovascular disease” (ICD-9-CM diagnosis codes 390.xx - 459.xx, excluding ischemic heart disease and hypertension), metabolic disorders, osteoporosis, and diabetes. The raloxifene cohorts had more patients with hypertension, diabetes, and dyslipidemia than the alendronate cohorts and differences were statistically significant in the 1-, 3-, and 5-year cohorts. Significantly higher pre-period prevalence of other cardiovascular disease, osteoporosis, rheumatoid arthritis and breast cancer was found among patients treated with alendronate. The number of patients having BMD screening was consistently lower in the raloxifene cohorts by a margin of 27.0 to 40.7 percentage points (p < 0.001). After applying the IPTWs, the differences in the majority of demographic and clinical variables became statistically insignificant between the raloxifene and alendronate cohorts. Most variables reached a desirable balance between cohorts as the standardized differences were below 10 [20]. A few exceptions included rural residence in the 7-year follow-up period and gastritis in the 8-year cohorts.

**Table 1:** **Unweighted patient demographic and baseline clinical characteristics**^**a**^

	**1-Year**		**3-Year**		**5-Year**		**6-Year**		**7-Year**		**8-Year**	
	**RLX**	**ALN**	**RLX**	**ALN**	**RLX**	**ALN**	**RLX**	**ALN**	**RLX**	**ALN**	**RLX**	**ALN**
Number of Patients	23,243	72,055	9,758	24,221	3,141	6,652	1,246	2,465	650	1,053	217	331
	63.0	64.9	63.3	65.2	63.0	65.3	62.9	64.7	62.3	64.3	60.7	63.2
Age (Mean, SD)	(9.5)	(10.7)*	(9.1)	(10.3)*	(8.8)	(10.0)*	(9.0)	(9.8)*	(8.8)	(9.6)*	(7.6)	(9.0)*
Insurance Plan Types^b^	*		*		*		*					
Comprehensive	43.0%	41.6%	50.0%	49.8%	55.8%	54.3%	55.9%	50.6%	60.9%	65.9%	54.4%	61.9%
Preferred provider organization	35.4%	33.9%	31.7%	29.6%	25.9%	23.8%	21.7%	19.1%	19.2%	14.5%	20.3%	11.8%
Point-of-service plan with capitation	6.1%	4.9%	7.4%	7.5%	11.3%	14.5%	17.9%	26.3%	16.3%	17.0%	23.5%	24.2%
Point-of-service plan	8.7%	6.9%	7.7%	5.7%	5.7%	4.3%	4.2%	3.8%	3.2%	2.5%	1.4%	1.8%
Health maintenance organization	6.1%	11.7%	3.0%	7.0%	1.1%	2.9%	0.3%	0.2%	0.3%	0.1%	0.5%	0.3%
Other/Unknown	0.6%	0.9%	0.2%	0.3%	0.1%	0.3%	0.0%	0.0%	0.0%	0.0%	0.0%	0.0%
Medicare Eligibility	38.1%	46.0%*	41.4%	49.3%*	41.8%	52.1%*	42.1%	50.8%*	39.4%	49.0%*	28.1%	40.8%†
Urban Residence	71.8%	80.5%*	69.0%	77.6%*	64.2%	71.6%*	54.3%	58.8%†	43.5%	37.4%†	28.6%	16.9%†
Charlson Comorbidity Index (Mean, SD)	0.46 (0.95)	0.54 (1.10)*	0.44(0.91)	0.48 (1.02)*	0.42 (0.87)	0.44 (0.97)	0.47 (0.92)	0.45 (0.98)	0.44 (0.87)	0.45 (0.99)	0.46 (0.87)	0.51 (1.32)
BMD Screening	44.4%	71.4%*	40.0%	69.9%*	33.4%	68.5%*	34.0%	70.3%*	31.1%	70.2%*	24.9%	65.0%*
Pre-period Fracture	3.9%	7.7%*	3.5%	6.8%*	2.8%	5.8%*	3.4%	6.2%*	2.9%	6.3%†	3.2%	5.7%
Confounding Conditions												
Hypertension	26.6%	25.2%*	27.4%	24.9%*	27.9%	24.6%*	28.4%	25.7%	28.0%	24.6%	29.0%	21.8%
Other Cardiovascular Disease	17.6%	20.0%*	17.3%	19.8%*	16.5%	19.6%*	15.5%	19.1%†	14.6%	19.6%†	13.4%	20.2%†
Metabolic Disorders	15.6%	14.9%†	15.1%	14.3%	15.0%	13.8%	13.6%	13.4%	14.2%	13.7%	17.1%	14.8%
Diabetes	9.2%	7.8%*	9.8%	7.0%*	8.9%	6.4%*	8.0%	6.1%†	8.3%	5.9%	8.8%	7.3%
Osteoporosis	9.4%	12.9%*	8.4%	13.2%*	7.6%	13.2%*	7.2%	13.8%*	7.8%	14.7%*	9.7%	16.3%†
Dyslipidemia	7.0%	6.2%*	6.9%	6.1%†	6.8%	5.7%†	6.4%	5.4%	7.1%	5.2%	8.3%	4.5%
Rheumatoid arthritis	1.7%	2.2%*	1.5%	2.2%*	1.1%	2.1%*	1.1%	2.4%†	1.2%	2.4%	1.4%	1.8%
Vasomotor symptoms	4.1%	3.5%*	4.3%	4.1%	4.1%	4.9%	3.8%	6.0%†	3.2%	7.3%*	1.4%	7.9%*
Ischemic heart disease	7.5%	7.5%	7.4%	7.6%	7.3%	7.6%	6.7%	7.2%	5.4%	6.6%	4.1%	6.9%
Breast cancer	4.8%	6.2%*	4.5%	5.8%*	5.0%	5.3%	5.9%	5.8%	5.4%	6.4%	5.5%	6.6%
Reflux	3.8%	2.7%*	3.7%	2.3%*	3.6%	2.5%†	3.6%	2.4%†	3.5%	2.1%	2.8%	2.1%
Gastritis	1.8%	1.4%*	1.5%	1.3%	1.4%	1.4%	0.9%	1.5%	1.1%	1.9%	0.5%	3.0%†
Gastric ulcer	0.4%	0.2%*	0.3%	0.2%	0.2%	0.2%	0.1%	0.1%	0.0%	0.2%	0.0%	0.3%
Peptic ulcer	0.1%	0.1%†	0.1%	0.1%	0.1%	0.1%	0.2%	0.1%	0.3%	0.2%	0.5%	0.0%
Confounding Medications												
Glucocorticoids	17.6%	19.1%*	16.4%	16.7%	16.0%	15.2%	15.0%	15.8%	13.1%	15.6%	12.0%	14.8%
Anticonvulsants	6.1%	6.6%†	5.3%	5.2%	4.4%	4.5%	4.1%	3.8%	4.0%	2.9%	3.2%	2.7%
Immunosuppressants	1.7%	3.1%*	1.4%	2.5%*	1.1%	2.3%*	0.7%	2.2%†	0.9%	2.7%†	0.9%	3.3%
Hormone Deprivation Therapy	2.2%	3.8%*	2.0%	3.5%*	2.4%	2.9%	2.8%	2.8%	2.5%	2.5%	2.8%	3.3%
Estrogen/Hormone Therapy	43.3%	29.7%*	50.3%	38.2%*	52.6%	45.5%*	38.0%	43.3%†	36.5%	42.4%†	36.4%	41.1%
Pre-period All-cause Healthcare Cost ($, Mean, SD)	6,954 (18,238)	7,544 (15,012)*	5,750 (12,698)	6,032 (12,196)	5,154 (9,073)	5,370 (11,866)	4,673 (7,807)	4,970 (9,188)	4,311 (6,449)	4,594 (6,652)	4,358 (7,198)	4,708 (6,952)
Pre-period Osteoporosis-related Healthcare Cost ($, Mean, SD)	268 (1,780)	484 (2,903)*	225 (1,394)	416 (2,738)*	226 (1,724)	337 (1,597)†	243 (2,626)	327 (1,581)	134 (573)	375 (2,121)†	173 (940)	319 (1,699)
Provider closest to Index Prescription												
Specialist	38.6%	37.5%†	38.9%	38.8%	39.4%	40.5%	43.1%	43.0%	39.8%	41.2%	38.7%	40.5%
Primary Care	17.0%	17.1%	15.9%	16.4%	15.7%	14.8%	14.2%	15.1%	14.2%	16.6%	13.4%	16.6%
Other/Unknown	44.4%	45.4%†	45.3%	44.8%	44.9%	44.8%	42.7%	42.0%	46.0%	42.2%	47.9%	42.9%

*P<0.001; †P<0.05.

^a^Due to limited space, not all covariates included in the IPTW and Multivariate models are presented in Table 1. Data can be provided upon request.

^b^Chi-Square test is used to test differences in insurance plan type between RLX and ALN cohort.

ALN=alendronate; BMD=bone mineral density; RLX=raloxifene; SD=standard deviation.

### Pre-period fractures

Prior to beginning treatment, a lower proportion of patients treated with raloxifene had a fracture compared to patients treated with alendronate. Differences in pre-period fracture rates ranged from 2.5 to 3.8 percentage points and these differences were significant with the exception of the 8-year cohorts (Table 1). After applying the IPTWs, the pre-period fracture rates for the raloxifene cohorts became higher than the alendronate cohorts in the 1-year (7.9%/raloxifene versus 6.8%/alendronate, p < 0.001), 3-year (7.5% versus 5.9%, p < 0.001), and 5-year (7.2% versus 5.0%, p < 0.001) follow-up period. The weighted pre-period fracture rates were not significantly different between patients treated with raloxifene and alendronate in the 6-year (6.3% versus 5.5%, p = 0.305), 7-year (4.5% and 5.1%, p = 0.563), and 8-year cohort (3.7% versus 5.0%, p = 0.455).

### Rate of vertebral and nonvertebral fracture

Before applying the IPTWs, patients treated with raloxifene appeared to have lower fracture rates during the treatment period than patients treated with alendronate. Patients with 1, 3, 5, and 7 years of adherent treatment with raloxifene had significantly lower rates of vertebral fracture than patients treated with alendronate. The patients treated with raloxifene had statistically significantly lower nonvertebral fracture rates in the 1- and 5-year follow-up periods (Table 2).

**Table 2:** Unweighted and weighted fracture rates in 1, 3, 5, 6, 7, 8 years of adherent treatment with RLX and ALN

**Length of followup**	**Before propensity weighting**							**After propensity weighting**						
	**RLX**			**ALN**			**P-value**	**RLX**			**ALN**			**P-value**
	**%**	**Lower 95% CI**	**Upper 95% CI**	**%**	**Lower 95% CI**	**Upper 95% CI**		**%**	**Lower 95% CI**	**Upper 95% CI**	**%**	**Lower 95% CI**	**Upper 95% CI**	
Vertebral Fracture														
1-year	0.19%	0.13%	0.25%	0.30%	0.27%	0.35%	0.002	0.34%	0.31%	0.38%	0.30%	0.27%	0.34%	0.334
3-years	0.67%	0.50%	0.83%	1.00%	0.89%	1.14%	0.002	0.93%	0.83%	1.03%	0.97%	0.87%	1.08%	0.720
5-years	0.92%	0.59%	1.26%	1.70%	1.40%	2.03%	0.002	1.48%	1.25%	1.71%	1.69%	1.43%	1.94%	0.451
6-years	1.20%	0.60%	1.81%	1.99%	1.44%	2.54%	0.083	1.28%	0.93%	1.64%	1.90%	1.46%	2.34%	0.169
7-years	1.38%	0.49%	2.28%	3.10%	2.08%	4.19%	0.024	2.14%	1.46%	2.82%	3.11%	2.29%	3.94%	0.231
8-years	0.92%	0.00%	2.19%	3.32%	1.39%	5.25%	0.071	3.70%	2.12%	5.27%	3.80%	2.19%	5.41%	0.952
Nonvertebral Fracture														
1-year	1.96%	1.78%	2.14%	2.20%	2.10%	2.31%	0.023	2.36%	2.27%	2.45%	2.15%	2.06%	2.24%	0.056
3-years	6.48%	5.99%	6.97%	6.80%	6.48%	7.12%	0.281	7.31%	7.04%	7.58%	6.70%	6.40%	6.93%	0.034
5-years	9.71%	8.67%	10.75%	11.80%	11.05%	12.61%	0.002	10.73%	10.14%	11.33%	11.48%	10.85%	12.12%	0.271
6-years	12.36%	10.53%	14.19%	13.18%	11.85%	14.52%	0.479	13.99%	12.89%	15.10%	12.99%	11.90%	14.07%	0.395
7-years	13.85%	11.19%	16.50%	15.29%	13.12%	17.46%	0.414	17.53%	15.74%	19.32%	15.29%	13.58%	17.01%	0.222
8-years	15.67%	10.83%	20.50%	18.73%	14.53%	22.93%	0.356	12.97%	10.16%	15.78%	18.82%	15.53%	22.12%	0.071

ALN=alendronate; RLX=raloxifene.

After applying the IPTWs, no statistically significant differences were found in the rates of vertebral fractures between the raloxifene and alendronate cohorts in all 6 time periods. There were also no statistically significant differences in the rates of nonvertebral fractures between the raloxifene and alendronate cohorts in each of the time periods, with the exception of the 3-year cohort (7.3%/raloxifene versus 6.7%/alendronate, p = 0.034).

### Risk of fracture

**Figure 2:**
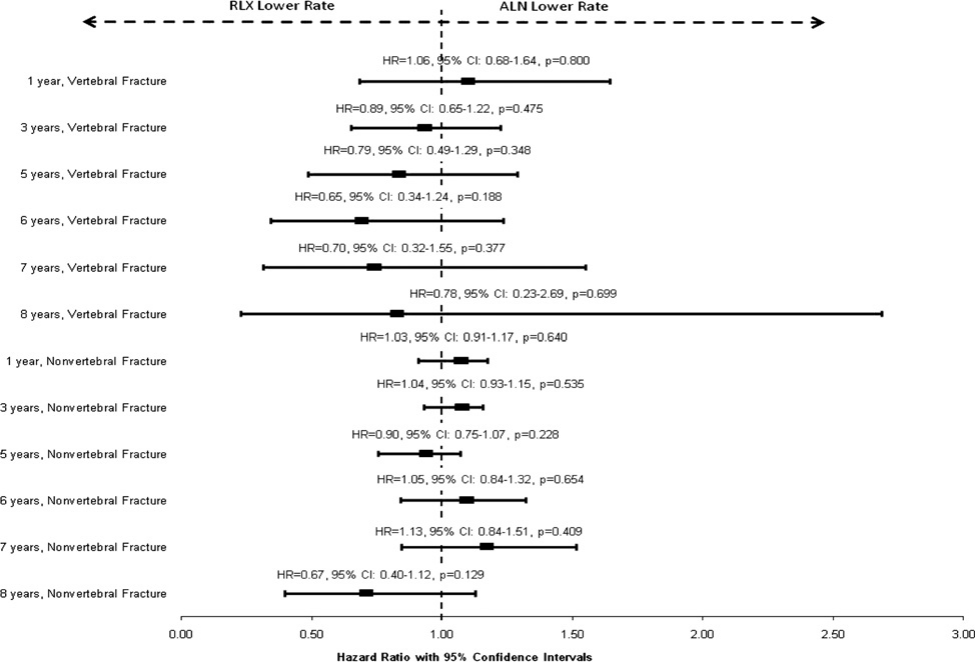
**Hazard ratios of RLX versus ALN for vertebral fracture and nonvertebral fracture in 1, 3, 5, 6 , 7 and 8-year cohorts, all patients (ALN served as the reference group).** For nonvertebral fracture, hazard ratios were fitted based on marginal models which adjusted for the clustering of multiple events. For vertebral fracture, hazard ratios were fitted based on standard Cox models. P>0.05 in all models. ALN=alendronate; HR=hazard ratio; RLX=raloxifene.

We examined the risk of fracture in the 6 observation periods by evaluating time to fracture as shown in Figure 2. The hazard ratios were not significantly different from 1 for vertebral fracture or for nonvertebral fracture in all six observation periods, indicating no significant difference in the risk of vertebral and nonvertebral fracture during 1, 3, 5, 6, 7,and 8 years of adherent treatment with raloxifene and alendronate.

The sensitivity analysis including only patients without pre-index fracture and examining the risk of vertebral and nonvertebral fracture in the 3-year cohort showed similar findings. There were no statistically significant differences in the risk of vertebral fracture (HR = 0.78, 95% CI: 0.56 – 1.09, p = 0.149) or nonvertebral fracture (HR = 1.06, 95% CI: 0.95 – 1.19, p = 0.287) during 3 years of adherent treatment with raloxifene or alendronate.

### Rate of breast cancer

**Figure 3:**
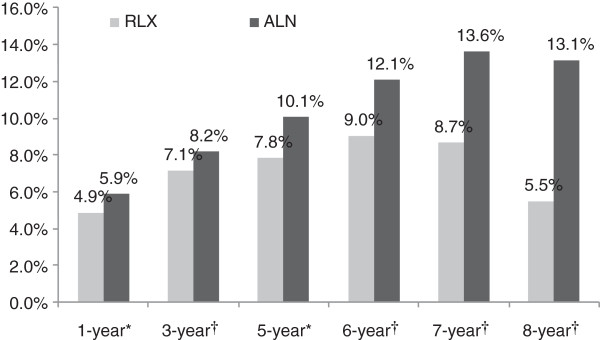
**Weighted breast cancer rates in 1, 3, 5, 6, 7, and 8 years of adherent treatment with RLX and ALN, all patients.** *P-value<0.001; †P-value<0.01. 95% confidence intervals: RLX: 1 year (4.75%-5.02%), 3 year (6.87%-7.41%), 5 year (7.31%-8.34%), 6 year (8.13%-9.95%), 7 year (7.34%-9.99%), 8 year (3.58%-7.38%); ALN: 1 year (5.73%-6.03%), 3 year (7.88%-8.47%), 5 year (9.46%-10.65%), 6 year (11.01%-13.11%), 7 year (11.98%-15.25%), 8 year (10.29%-15.99%). ALN=alendronate; RLX=raloxifene.

Rates of breast cancer in the raloxifene cohorts were significantly lower than in the alendronate cohorts. The absolute difference in rates increased with increasing duration of follow-up. In 1, 3, 5, 6, 7, and 8 years of treatment, the unweighted breast cancer rates were 1.6 (p < 0.001), 1.8 (p < 0.001), 2.3 (p < 0.001), 2.1 (p = 0.051), 3.9 (p = 0.015), and 6.5 (p = 0.018) percentage points lower for the raloxifene cohorts than the alendronate cohorts, respectively. Figure 3 displays the breast cancer rates weighted by applying IPTWs. The weighted rates were significantly lower in raloxifene cohorts by 1.0-7.6 percentage points. In the sensitivity analyses, which excluded pre-existing breast cancer cases, differences in the 1- and 3-year cohorts in rate of breast cancer were not observed. However, significantly lower breast cancer rates were found in the raloxifene cohorts compared to the alendronate cohorts in the longer follow-up periods. The risk of newly diagnosed breast cancer, weighted using the IPTWs, was 1.6 (p < 0.001), 3.0 (p < 0.001), 3.7 (p = 0.002), and 5.6 (p = 0.004) percentage points lower among the raloxifene recipients than their alendronate counterparts in the 5-, 6-, 7-, and 8-year follow-up cohorts, respectively.

## Discussion

This study examined fracture risk reduction during adherent use of raloxifene and alendronate in claims data. Patients treated with raloxifene were younger, had a lower pre-period incidence of fracture, and had a lower incidence of osteoporosis diagnosis than patients treated with alendronate. After adjusting for baseline demographic and clinical characteristics by applying the IPTWs, the rates of vertebral and nonvertebral fracture were not significantly different between the patients treated with alendronate and raloxifene in most of the follow-up periods examined. In the 3-year cohort, there was no significant difference in vertebral fracture but a significant difference in nonvertebral fracture (7.3%/raloxifene versus 6.7%/alendronate, p = 0.034) was observed. Once time-to-fracture was taken into consideration, no significant difference in the risk of vertebral or nonvertebral fracture was found in all 6 observation periods. In addition, a similar result was observed when restricting to patients without pre-index fracture in the 3-year cohort. These results suggest that patients experienced a similar reduction in fracture risk during treatment with raloxifene and alendronate.

Randomized clinical trials (RCTs) and claims based retrospective studies examining the effectiveness of bisphosphonates and raloxifene have been published [5-7,9,21-23]. The Multiple Outcomes of Raloxifene Evaluation (MORE) trial found the relative risk of nonvertebral fracture (excluding traumatic fractures) for patients treated with raloxifene was not significantly different from patients receiving placebo after 3 years (RR = 0.9, 95% CI: 0.8 - 1.1) [7] and 4 years of observation (RR = 0.93, 95% CI: 0.81 - 1.06) [23]. In the Fracture Intervention Trial (FIT) of the effect of alendronate, the relative risk of nonvertebral fracture (excluding traumatic fractures) was 0.88 (95% CI: 0.74 - 1.04) among patients without a baseline vertebral fracture at 4 years [6] and 0.80 (95% CI: 0.63 - 1.01) among patients with a baseline vertebral fracture at 3 years [5]. In a double-blind, randomized comparison of patients treated with alendronate or raloxifene, Recker and colleagues examined postmenopausal women with a femoral neck BMD T-score between −2.5 and −4.0, inclusive, no prevalent vertebral fractures, and no prior bone-active agent use [11]. Although the trial was stopped early, resulting in insufficient power to show non-inferiority between therapies, the primary endpoint of ≥1 new osteoporotic vertebral or nonvertebral fracture occurred in 22/713 alendronate (3.1%) patients and 20/699 raloxifene (2.9%) patients.

Cadarette and colleagues published an insurance claims-based cohort study of 43,135 patients aged at least 65 years who initiated oral bisphosphonates, nasal calcitonin, or raloxifene while enrolled in 2 statewide pharmaceutical benefit programs [21]. The mean age was 79 years and 96% were women. The primary outcome was nonvertebral fracture (hip, humerus, or radius or ulna) within 12 months of treatment initiation. Similar to our findings, significant differences in fracture risk were not found between alendronate, risedronate, and raloxifene. However, among those with a fracture history, patients treated with raloxifene experienced more nonvertebral fractures within 12 months (HR = 1.78, 95% CI: 1.20 to 2.63) compared with those treated with alendronate.

The Cadarette et al. study included Medicare enrollees from 2 states with an average age of 79 years, whereas our study included patients with both commercial and Medicare supplemental insurance, with an average age of 61 to 65 years old. In the Cadarette et al. study, an intent-to-treat approach was used and patients were only required to receive treatment with raloxifene or bisphosphonate at index. Our study took an on-treatment approach and required a minimum MPR of 80% of index medication in each of the follow-up periods. Additionally, patients were required not to switch to another osteoporosis medication during the follow-up period. Our study examined fracture rates during longer treatment periods including 3-, 5-, 6-, 7-, and 8-year cohorts. In addition to nonvertebral fracture, we also evaluated the rate of vertebral fracture and found no significant differences between adherent treatment of raloxifene and alendronate. Our sensitivity analysis based on only patients without pre-index fracture confirmed that there was no significant difference in the risk of vertebral or nonvertebral fracture after 3 years of adherent treatment with raloxifene or alendronate.

Raloxifene has been shown to reduce the risk of invasive breast cancer in postmenopausal women with osteoporosis and in postmenopausal women at high risk for invasive breast cancer [9,10]. Recent analysis has suggested that bisphosphonates might also reduce the risk of breast cancer [24]. In this study, the rate of breast cancer was lower in the raloxifene group compared with the alendronate group and the gap between the groups increased in the cohorts with longer duration of follow-up.

Consistent with previous studies, [21,25] we found that patients treated with raloxifene were younger, less likely to be diagnosed with osteoporosis and have BMD screening, and had lower rates of pre-period fracture than those treated with alendronate. We used IPTW methodology to adjust for these and other confounders and achieved cohort balance after weighting. Due to the limitations of administrative claims data, we were unable to adjust for clinical risk factors such as BMD score. Only clinically-diagnosed fractures recorded in medical claims were analyzed. We included closed fracture, pathological, and stress fracture as a proxy of osteoporosis-related fracture but we were unable to review patients’ medical records to validate this assumption. Due to the difficulty of identifying re-fracture of the same fracture site using medical claims, we only reported the first fracture at a given fracture site. This is a conservative approach with inherent underestimation of the fracture rate. Patients were required to be eligible for insurance and adherent with their medication during the fixed-length period and could not switch to another study medication. This could potentially underestimate the fracture rate, but the same criteria were applied to both treatment groups. This study was not designed to specifically evaluate reduction of breast cancer risk thus we did not specifically control for risk factors such as family history of breast cancer. Additionally, the sample sizes of longer follow-up length cohorts were small which should be taken into consideration when evaluating these findings. Finally, these findings may not be generalizable to populations outside of those evaluated in this study.

## Conclusions

Fracture is the major consequence of osteoporosis and prevention of fracture is central to cost containment in the management of osteoporosis. In this study, raloxifene- and alendronate-treated patients had similar weighted fracture rates, and raloxifene-treated recipients had lower rates of breast cancer.

## Competing interests

Funding for the study was provided by Eli Lilly and Company. SAF, RB and JHK are employees and stockholders of Eli Lilly and Company. JS is a former employee of Eli Lilly and Company and holds shares of stock in Eli Lilly and Company. SC, NS, BC, and DRD are all employees of Truven Health Analytics. Truven Health Analytics received funding from Eli Lilly and Company to conduct this analysis.

## Authors’ contributions

SAF, JHK, JS, RB, and SC initiated the study and developed the conceptual research design for the analysis. SC led the study and reviewed the manuscript. SAF, JHK, JL, and RB made important intellectual contributions and reviewed the manuscript. NS contributed to the study design, data analysis and drafted the report and manuscript. NS, BC and DRD provided computer programming, statistical analyses and manuscript reviews. All authors approved the final version of the manuscript.

## Financial support information

This study was fully funded by Eli Lilly and Company.

## Pre-publication history

The pre-publication history for this paper can be accessed here:

http://www.biomedcentral.com/1472-6874/13/15/prepub
